# Non-Clinical Autistic Traits Correlate With Social and Ethical but Not With Financial and Recreational Risk-Taking

**DOI:** 10.3389/fpsyg.2020.00360

**Published:** 2020-03-11

**Authors:** Kristel De Groot

**Affiliations:** ^1^Institute of Psychology, Erasmus School of Social and Behavioural Sciences, Erasmus University Rotterdam, Rotterdam, Netherlands; ^2^Department of Applied Economics, Erasmus School of Economics, Erasmus University Rotterdam, Rotterdam, Netherlands; ^3^Erasmus University Rotterdam Institute for Behaviour and Biology, Erasmus University Rotterdam, Rotterdam, Netherlands

**Keywords:** autism spectrum disorder, Autism-Spectrum Quotient, Domain-Specific Risk-Taking scale, uncertainty, risk

## Abstract

Previous research into uncertain and risky decision-making in autism spectrum disorder (ASD) has been inconclusive, with some studies reporting less uncertain and risky decisions by persons with ASD compared to neurotypicals, but other studies failing to find such effects. A possible explanation for these inconsistent findings is that aberrant decision-making in ASD is domain-specific, and only manifests itself in domains related to autism symptomatology. The present study examines this premise by correlating self-reported autistic traits to individuals’ intention to engage in risky behaviours, their perception of how risky these behaviours are, and the amount of benefit they expect to obtain from engaging in them; all for five separate domains of decision-making: social, ethical, recreational, health/safety, and financial. In line with the hypotheses, persons with higher autistic traits reported reduced intention to engage in risky social behaviours and increased intention to engage in risky ethical behaviours. Furthermore, a positive correlation was found between autistic traits and risk perception in the social domain, indicating that persons with higher autistic traits perceive social behaviours as riskier than do persons with lower autistic traits. Correlations between autistic traits and individuals’ intention to engage in risky recreational and financial behaviours were small, and supported the null hypothesis (as shown by Bayes Factors). Given that most studies on uncertain and risky decision-making take place in a financial context, the present results could explain previous inconsistent findings on decision-making in ASD. Therefore, future studies should also examine decision-making outside the financial realm.

## Introduction

Decision-making is a key process that can have major consequences for personal and professional life. Hence, research into individuals’ decision processes not only contributes to our understanding of human cognition, but can also have considerable practical implications. In addition to studying decision-making in typical populations, some investigations focus on clinical ones. One such group concerns individuals with autism spectrum disorder (ASD), a neurodevelopmental syndrome characterised by difficulties in social communication, restricted and repetitive behaviour, a preference for sameness and routines, and sensory abnormalities (American Psychiatric Association, 2013). Examining decision-making in persons with ASD may identify situations in which their decision-making is impeded, as well as situations in which their decisions outperform those of ‘neurotypical’ individuals.

For most decisions we make, it is unclear what the exact outcome will be. Many situations are therefore characterised by risk (where we know the probabilities associated with the outcomes) or uncertainty (where these probabilities are unknown) (Knight, 1921). Research into such uncertain or risky decision-making in ASD has so far been inconclusive. For example, some studies show that individuals with ASD make less uncertain and/or risky decisions (De Martino et al., 2008; South et al., 2014; Levin et al., 2015), whereas others fail to find such differences between individuals with and without ASD (Johnson et al., 2006; South et al., 2008, 2011).

A possible explanation of these conflicting findings is that aberrant decision-making in ASD is domain-specific. Levin et al. (2015) suggest that especially social uncertainty/risk may be salient for persons with ASD, and that it would therefore be useful to examine domains separately. This premise fits with autism symptomatology: given that persons with ASD show difficulties in social interaction and communication (American Psychiatric Association, 2013), it is reasonable to expect aberrant decision-making in the social domain. In contrast, autism symptomatology does not suggest that aberrant decision-making is likely to occur in, for instance, the financial domain. Since most studies examining uncertain and risky decisions employ financial incentives (Johnson et al., 2006; De Martino et al., 2008; South et al., 2008, 2011, 2014; Levin et al., 2015), this may explain the lack of consistent findings on decision-making in individuals with ASD.

To address the premise that decision-making in ASD only differs from neurotypical decision-making in a subset of domains, Levin et al. (2015) suggest using the Domain-Specific Risk-Taking (DOSPERT) scale, a self-report measure that assesses risky^1^ decision-making across five domains: social, ethical, recreational, health/safety, and financial (Blais and Weber, 2006). To the knowledge of the author, so far only one study has used the DOSPERT scale in relation to ASD. Gaeth et al. (2016) found that individuals with ASD reported reduced intention to engage in risky social behaviours and increased intention to engage in risky ethical behaviours, compared to a control group. No differences were observed for any of the other domains, supporting the premise that aberrant decision-making in ASD is domain-specific.

The present study extends the findings by Gaeth et al. (2016) in two ways. First, it aims to replicate the findings in the broader autism spectrum, that is, along a continuum ranging from individuals who report few autistic traits to individuals who report many (Wing, 1988; Baron-Cohen et al., 2001). Previous research on this broader spectrum has demonstrated that clinical characteristics are (to a lesser extent) also observed in the general population (De Groot and Van Strien, 2017). The present study examines whether this holds for risk-taking too. Second, Gaeth et al.’s (2016) findings are extended by including not one but all three DOSPERT scales. In addition to the scale examining individuals’ intention of engaging in risky behaviours (risk-taking), the DOSPERT includes two additional scales: one asking individuals how risky they think each behaviour is (risk perception), and one asking them how much benefit they think they would obtain from engaging in it (expected benefit). Data from these two additional scales may clarify why individuals show certain levels of risk-taking on the first scale.

Specifically, the present study examines the correlations between autistic traits and individuals’ (1) risk-taking, (2) risk perception, and (3) expected benefit for activities/behaviours in five separate decision domains: social, ethical, recreational, health/safety, and financial. Based on Gaeth et al.’s (2016) findings, it is hypothesised that autistic traits correlate negatively with the intention to engage in risky social behaviours, and positively with the intention to engage in risky ethical behaviours. Furthermore, it is expected that individuals’ intention to take risks in other domains is not correlated with autistic traits. Since the former hypotheses favour *H1* and the latter favour *H0*, Bayes Factors are computed in order to quantify evidence for both *H1* and *H0* (Wagenmakers et al., 2016; Quintana and Williams, 2018). Given the lack of research on risk perception and expected benefit in relation to ASD, no hypotheses are formulated for these constructs.

## MATERIALS AND METHODS

### Participants

The participants were 240 students. The sample was approximately gender-balanced, consisting of 113 male and 127 female participants with a mean age of *M* = 20.88 (SD = 3.32), range 17–55 years. Most participants were studying social sciences (53.33%), management (17.08%), or economics (15.83%). The study was in accordance with the Helsinki declaration, and received ethics approval from the Erasmus Research Institute of Management (ERIM) review board. All participants provided written informed consent.

### Measures

#### Autism-Spectrum Quotient

The Autism-Spectrum Quotient (AQ) (Baron-Cohen et al., 2001) is a self-report measure of autistic traits in adults of normal intelligence, and consists of 50 items that are answered on a 4-point Likert-scale. Contrary to Baron-Cohen et al. (2001), all four answer options were included in scoring as this provides better results on psychometric indices (Murray et al., 2016; Stevenson and Hart, 2017). Cronbach’s alpha was α = 0.82.

#### Domain-Specific Risk-Taking Scale

The full DOSPERT scale (Blais and Weber, 2006) consists of three scales that each contain the same 30 items, divided into five subscales: social, ethical, recreational, health/safety, and financial. Each item describes a risky activity/behaviour, such as disagreeing with an authority figure (social), leaving your children alone while running an errand (ethical), or betting one’s income in sports (financial). On scale one, individuals rate their likelihood of engaging in each risky behaviour (risk-taking); on scale two, they rate their gut level assessment of the riskiness of each behaviour (risk perception); and on scale three, they rate how much benefit they think they would obtain from engaging in each behaviour (expected benefit). All items are answered on a 7-point Likert-scale. Cronbach’s alpha for the three composite scales was α = 0.88, α = 0.86, and α = 0.85, respectively; and α = 0.66–0.85, α = 0.72–0.87, and α = 0.51–0.89 for the subscales.

### Procedure

The data were collected as part of two separate studies (*n* = 124 and *n* = 116) on decision-making under uncertain and risky conditions. The questionnaires were filled out online by participants, who were urged to do so in a quiet environment.

### Analyses

First, the possibility of Common Method Bias (CMB) was examined because all measures used in the present study are of the same type (self-report). To this end, one (unrotated) factor was extracted from a principal component analysis in order to examine how much variance that one factor explained. Second, possible Insufficient Effort Responding (IER) was examined by checking response times (RTs) under the assumption that short RTs indicate limited cognitive processing (Huang et al., 2012). Third, descriptive statistics for the AQ and the three DOSPERT scales were computed. Fourth, the hypotheses were tested by correlating the AQ score with the first, second, and third DOSPERT scale (risk-taking, risk perception, and expected benefit, respectively). Because some predictions favoured *H0*, frequentist statistics were deemed unsuitable, and Bayes Factors (BFs) rather than *p*-values were reported.^2^

BFs are an implementation of Bayesian hypothesis testing, and quantify the degree to which data favour *H1*, *H0*, or neither hypothesis (Wagenmakers et al., 2016; Quintana and Williams, 2018). It is a relative measure that compares the predictive accuracy of the two models. BFs can thus indicate that data are more likely to have occurred under one model than another, but do not specify the absolute performance of a model. Therefore, it is still valuable to report the absolute correlation size, with Pearson’s *r* = 0.10 indicating a small, 0.30 indicating a medium, and 0.50 indicating a large effect (Cohen, 1992). BFs were interpreted using the classification of Jeffreys (1961) as adjusted by Lee and Wagenmakers (2013), and were computed with the open-source software program JASP (JASP Team, 2018). Computing BFs requires specifying a prior distribution, which is based on prior knowledge (often from previous studies) about the parameters of the model. Given the scarcity of previous research on the topic examined here, the JASP default (a stretched beta prior width of 1) was used, which only limits ρ to values between −1 and +1. The impact of the prior on the results was examined in a robustness check. A second robustness check examined the impact of excluding participants with psychiatric or neurological disorders. A third robustness check examined the impact of conceptual overlap between items from the AQ and the DOSPERT.

## Results

### Common Method Bias

All items were loaded into a principal component analysis from which one factor was extracted. This was done for the AQ items together with those of each of the DOSPERT scales. A single factor explained 11.02% of variance for the AQ and risk-taking items, 9.66% for the AQ and risk perception items, and 9.24% for the AQ and expected benefit items. Since the single factor could not account for a majority of variance, it was concluded that CMB was not an issue.

### Insufficient Effort Responding

Response times were available for one of the subsamples (*n* = 116). Since the survey allowed participants to temporarily abandon and later return to filling out the questions, a few extreme RTs of several hours were observed, which heavily impacted the mean and SD. Therefore, RTs ≥ 60 min were discarded, leaving *n* = 111. The average RT of this trimmed sample was *M* = 16.86 (SD = 8.37), range 5.62–54.72 min. Six participants scored ≥1 SD below the mean. Inspection of their data did not reveal any signs of IER: none had odd response patterns, and their average scores on the 19 study variables only significantly differed from those of the other participants once (which can be expected based on a 5% chance level).

### Descriptive Statistics

The average AQ score was *M* = 108.42 (SD = 13.33, range 71–156). The DOSPERT average total scores were *M* = 107.30 (SD = 24.04, range 50–162) for risk-taking, *M* = 127.58 (SD = 21.27, range 73–183) for risk perception, and *M* = 100.43 (SD = 20.33, range 47–165) for expected benefit. Independent *t*-tests showed that men scored higher than women on autistic traits [*t*(238) = 2.47, *p* = 0.014, $\eta_{p}^{2}=0.02$], risk-taking [*t*(238) = 6.19, *p* < 0.001, $\eta_{p}^{2}=0.14$], and expected benefit [*t*(238) = 3.62, *p* < 0.001, $\eta_{p}^{2}=0.05$]. Women scored higher than men on risk perception [*t*(238) = −4.86, *p* < 0.001, $\eta_{p}^{2}=0.09$].

Visual inspection of the histograms and normal *Q*–*Q* plots indicated that all full scales were approximately normally distributed. This was supported by quantitative distribution indices: skewness was 0.02 for autistic traits, −0.03 for risk-taking, −0.04 for risk perception, and −0.23 for expected benefit (SE = 0.16); kurtosis was 0.53 for autistic traits, −0.44 for risk-taking, −0.16 for risk perception, and 0.10 for expected benefit (SE = 0.31). Four DOSPERT subscales were skewed: ethical and financial risk-taking, and health/safety expected benefit were right skewed; financial risk perception was left skewed. Inspection of suspected outliers showed that these resulted from persons having one or more extreme but plausible subscale scores. Therefore, all participants were retained in the analyses.

### Correlation Analyses

The correlations between autistic traits (AQ) and the three DOSPERT scales are presented in Table 1 for each subscale of the DOSPERT separately.

**TABLE 1:** Correlations between autistic traits (AQ) and the three DOSPERT scales for each subscale of the DOSPERT separately.

		**Correlation**		
		**AQ, DOSPERT risk-taking**	**AQ, DOSPERT risk perception**	**AQ, DOSPERT expected benefit**
**DOSPERT subscale**				
Social	*r*	−0.29	0.32	−0.05
	BF10	3696.05	28726.13	0.11
Ethical	*r*	0.22	−0.06	0.12
	BF10	34.94	0.13	0.51
Financial	*r*	0.08	−0.03	0.08
	BF01	6.18	11.38	5.38
Recreational	*r*	−0.06	−0.02	−0.03
	BF01	7.91	11.83	11.44
Health/safety	*r*	−0.12	−0.08	0.04
	BF01	2.00	5.98	9.92
Total	*r*	−0.04	0.03	0.06
	BF01	9.89	11.19	7.90

*Bayes Factors are a relative measure and provide a ratio between the likelihood of the null and that of the alternative model. This ratio can be reported as BF10 (evidence for *H*1 relative to *H*0) or BF01 (evidence for *H*0 relative to *H*1), which are each other’s inverse: BF10 = 1/BF01. Since previous research (Gaeth et al., 2016) reported effects in the social and ethical domain, for these domains BF10 was reported. Since the hypotheses predicted no effects for the other domains, BF01 was used for those. BFs were interpreted using the classification of Jeffreys (1961) as adjusted by Lee and Wagenmakers (2013), in which BF10 = 1–3 (BF01 = 1/3−1) is considered anecdotal evidence; BF10 = 3–10 (BF01 = 1/10−1/3) moderate evidence; BF10 = 10–30 (BF01 = 1/30−1/10) strong evidence; BF10 = 30–100 (BF01 = 1/100−1/30) very strong evidence; and BF10 > 100 (BF01 < 1/100) extreme evidence.*

As hypothesised, autistic traits were negatively correlated with individuals’ intention to engage in risky social behaviours (*r* = −0.29) and positively with their intention to engage in risky ethical behaviours (*r* = 0.22). The BFs indicated that the evidence in support of *H1* was ‘extreme’ for social behaviours and ‘very strong’ for ethical ones, with the data being respectively almost 3700 and 35 times more likely to have occurred under *H1* than *H0*. For the financial, recreational, and full score, correlations were small (0.08, −0.06, and −0.04, respectively), and the BFs indicated ‘moderate’ evidence for *H0*. Evidence for the health/safety domain was ‘anecdotal’ (*r* = −0.12, BF01 = 2.00), hence supporting neither hypothesis.

Regarding the correlations between autistic traits and risk perception, the data again showed ‘extreme evidence’ for *H1* in the social domain, with individuals with higher autistic traits judging social behaviours as riskier (*r* = 0.32, BF10 = 28726.13). The correlations between autistic traits and the other domains plus the full score were smaller (−0.06, −0.03, −0.02, −0.08, 0.03), and their accompanying BFs provided support for *H0* that was of either ‘moderate’ (ethical, health/safety) or ‘strong’ (financial, recreational, full score) magnitude.

Finally, for the expected benefit scale, none of the correlations favoured the *H1*. For the recreational subscale (*r* = −0.03) the BF provided ‘strong’ evidence for *H0*. The *H0* received ‘moderate’ evidence for the social, financial, health/safety, and full score (correlations −0.05, 0.08, 0.04, and 0.06, respectively), and ‘anecdotal’ evidence for the ethical subscale (*r* = 0.12).

### Robustness Analyses

Three robustness checks were run. The exact findings of these robustness analyses are reported in the Supplementary Material.

First, given the sensitivity of BFs to the prior (Kass and Raftery, 1995), the impact of assigning different priors was examined. BFs were examined for prior widths ranging from approximately 0.25 to 2 (disregarding widths around 0, given that they are unrealistically small and almost always result in a BF of 1). Across this range, over half of the BFs changed from moderate to strong magnitude, or vice versa. This was to be expected, given that many of the initial BFs fell around this interpretation line (BF10 = 10 or BF01 = 1/10). Because of this, some of the other BFs also moved one classification category up or down. Overall, none of the main conclusions changed.

A second robustness check examined the impact of excluding participants with a current psychiatric or neurological disorder. Given the present focus on traits that are assumed to exist on a population-wide spectrum, these individuals were included in the main analyses since they are also assumed to be on that spectrum. However, since psychiatric or neurological disorders may impact a person’s autistic trait level, the results were examined after excluding these individuals. Based on self-reported diagnosis, 15 participants were excluded (one of whom reported ASD). No major changes in the correlation coefficients or the BFs were observed.

A third robustness check examined the impact of conceptual overlap between items from the AQ and the DOSPERT. In the main analyses, the full AQ score was used since previous literature did not allow for more specific hypotheses (focused on subsets of autistic traits). However, it is possible that the correlations between the AQ and the DOSPERT social scores were inflated as a result of conceptual overlap between these DOSPERT items and AQ items focused on the social characteristics of autism. Re-running the analyses using an AQ score based on only 40 items (disregarding the 10 items from the AQ ‘social skill’ subset) indeed lowered the correlations and accompanying BFs for the social subscale of both risk-taking and risk perception. However, the correlations remained of moderate size (from *r* = −0.29 to *r* = −0.25; and from *r* = 0.32 to *r* = 0.29), and the BFs still provided ‘extreme’ evidence for *H1* (from BF10 = 3696.05 to BF10 = 134.72; and from BF10 = 28726.13 to BF10 = 2647.94).

## Discussion

The present study examined the correlations between autistic traits and individuals’ intention of engaging in risky behaviours, their perception of how risky these behaviours are, and the amount of benefit they expect to obtain from engaging in these behaviours. Correlations were examined for behaviours in five separate domains of decision-making, of which (based on previous work) especially the social, ethical, and financial domain were of interest to the present study. In line with the hypotheses, individuals scoring higher on autistic traits reported a lower intention to engage in risky social behaviours and a higher intention to engage in risky ethical behaviours. Bayes Factors indicated that for both correlations the data favoured the*H*1 over the *H0*. In contrast, the correlation between autistic traits and the intention to engage in risky financial behaviours was small, and favoured the *H0*. Most correlations between autistic traits and the other two DOSPERT scales (risk perception and expected benefit) were also small, and again favoured the *H0*. A notable exception was the positive correlation between autistic traits and social risk perception, for which the data were better explained by the *H1*.

For the social domain, both the reduced risk-taking and the increased risk perception may be explained by social motivation theory (Chevallier et al., 2012). If individuals with higher autistic traits find social activities less rewarding, they may be less inclined to engage in such activities (resulting in decreased social risk-taking). Since they then gain less experience with these activities, they may become more wary of them (hence increased perception of risks). An alternative explanation can be found within the DOSPERT framework itself, as the positive correlation between autistic traits and the second DOSPERT scale (risk perception) could explain the negative correlation between autistic traits and the first DOSPERT scale (risk-taking): individuals with higher autistic traits might engage less in risky social behaviours because they perceive such behaviours as riskier than do individuals with lower autistic traits. This premise was supported by an ex post mediation analysis showing that for the social domain, risk perception indeed mediated the relationship between AQ and risk-taking [β = −0.09, SE = 0.03, CI (−0.15, −0.04)]. Thus, the relationship between individuals’ autistic traits and their intention to engage in risky social behaviours can in part be explained by how they perceive these risks.

In contrast to the social domain, the positive correlation between autistic traits and the intention to engage in risky ethical behaviours could not be explained by data from the additional DOSPERT scales, since these latter correlations favoured the *H0*. Alternatively, this positive correlation may be explained by the assertion that individuals with higher autistic traits incorporate less emotional information in their decision-making and hence make more rational decisions, as demonstrated by their reduced sensitivity to framing effects (Shah et al., 2016), composition effects (Farmer et al., 2017), and ownership effects (Hartley and Fisher, 2018). This may especially impact decisions in the ethical domain, since one’s emotional response towards a situation is thought to influence judgement of the ethical acceptability of that situation (Brewer et al., 2015). This more rational decision style could explain the increased intention of individuals with high autistic traits to engage in risky ethical behaviours, despite not perceiving these behaviours as less risky or as yielding more benefit. It also fits with findings on moral decision-making by individuals with autism, who more frequently opt for utilitarian decisions in personal moral dilemmas than neurotypicals do (Gleichgerrcht et al., 2013). Presented with such dilemmas, they show reduced emotional reactions, despite having similar perceptions of the appropriateness of the judgements. Moreover, individuals with autism who make utilitarian choices show a decreased ability to infer other people’s intentions and to take their perspective. Hence, making more rational ethical decisions may also result from poorer cognitive empathy.

Finally, an explanation for the null findings in the financial domain has already been proposed in the introduction, where it was argued that autism symptomatology is not indicative of aberrant financial risk-taking, and that studies solely focusing on the financial domain may therefore not find a consistent relationship between risk-taking and autistic traits. Together with Gaeth et al.’s (2016) findings in individuals with clinically diagnosed ASD, the present study supports this premise, and stresses the importance of examining decision-making in non-financial domains. In addition to self-report scales like the DOSPERT, the feasibility of this recommendation has been shown for behavioural risk tasks, which have sparsely but successfully been used with non-financial incentives, such as social rewards (Op de Macks et al., 2017). Other tasks, like the Prisoner’s Dilemma Game, the Dictator Game, and the Ultimatum Game, also incorporate social elements in the decision process, and have been shown to work well in relation to autism (see e.g. Sally and Hill, 2006). Future studies should follow-up on that and look beyond financial decision-making, especially when examining clinical groups or associated traits for which aberrant financial decisions are not naturally expected based on existing knowledge of their symptomatology.

## Data Availability Statement

The datasets collected for this study are available on request to the corresponding author.

## Ethics Statement

The study was in accordance with the Helsinki declaration, and received ethics approval from the Erasmus Research Institute of Management (ERIM) review board. All participants provided written informed consent.

## Author Contributions

KG conceived of the manuscript, collected and analysed all data, and wrote up this final report.

## Conflict of Interest

The authors declare that the research was conducted in the absence of any commercial or financial relationships that could be construed as a potential conflict of interest.

## Footnotes

1 The DOSPERT scale is presented as measuring intentions, perceptions, and expectations regarding risky behaviours (Blais and Weber, 2006). However, a recent article (De Groot and Thurik, 2018) makes the case that the behaviours included in the scale are characterised by uncertainty rather than risk, since the probabilities associated with the outcomes of the behaviours are unknown (Knight, 1921). In the present article, the term ‘risk’ is used in relation to (findings based on) the DOSPERT scale, in order to stay true to the original literature, and to prevent confusion among readers already familiar with the scale. Nevertheless, readers should bear in mind that participants’ answers are given under conditions of uncertainty.

2 The assertion that frequentist statistics can only reject and not support *H0*, is actually flawed. In frequentist statistics, the *p*-value of a hypothesis can indeed only reject the null hypothesis. In other words, the *p*-value of *H0* cannot accept *H0*. However, *H0* does not need to be zero. Defining *H0* as an effect ‘big enough to be of interest’, one can reject the null that an effect is big enough to be of interest, and thus conclude that the effect is nearly zero (Hodges and Lehmann, 1954; Cohen, 1988; Rindskopf, 1997). The *H1* then states that the effect (for example, the correlation) between *X* and *Y* is very small (negligible). The *H0* then states that the effect is large enough to be deemed important. Now the *H0* and *H1* are ‘composite’ hypotheses (both consisting of a range of possible values) rather than ‘point’ hypotheses (single values). This way, one could for example test the *H1* that the correlation between *X* and *Y* is less than *r* = 0.05 (very small) against the *H0* that the correlation is at least *r* = 0.05 by asking whether *H1* (*r* < 0.05) lies in the confidence interval (CI) and *H0* (*r* ≥ 0.05) lies outside of it. If the CI includes the negligible effect and excludes all values big enough to be of interest, one can accept the null. With this approach, the present study could have used frequentist statistics, though using BFs is more straightforward here.
