# Supplementary material for: Non-Clinical Autistic Traits Correlate With Social and Ethical but Not With Financial and Recreational Risk-Taking
Source: Front Psychol. 2020 Mar 11;11:360. doi: 10.3389/fpsyg.2020.00360 (PMC7078360; doi:10.3389/fpsyg.2020.00360)
Supplement: Supplementary file 1 [file Data_Sheet_1.PDF]

Supplementary Material

1 Robustness check – Impact of the Prior

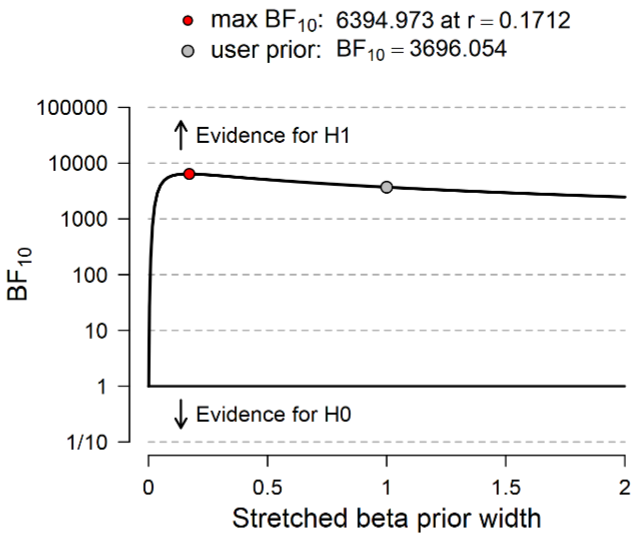

Supplementary Figure 1. Bayes Factor robustness check on the correlation between social risk-taking and autistic traits.

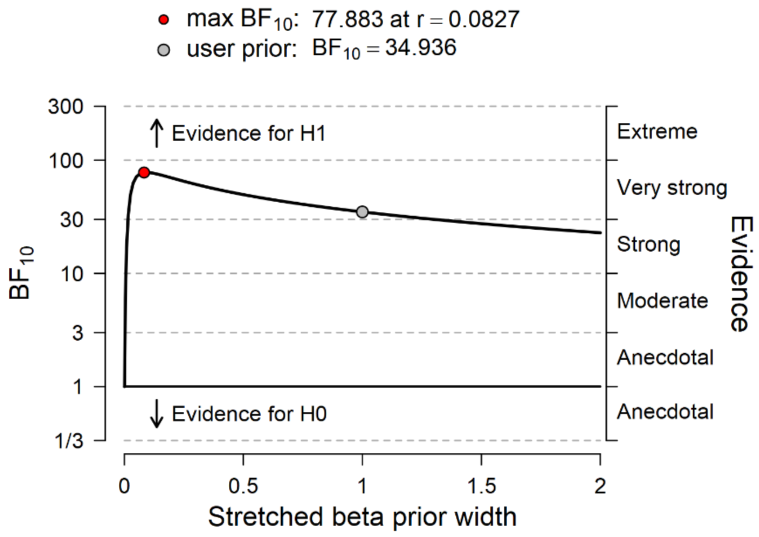

Supplementary Figure 2. Bayes Factor robustness check on the correlation between ethical risk-taking and autistic traits.

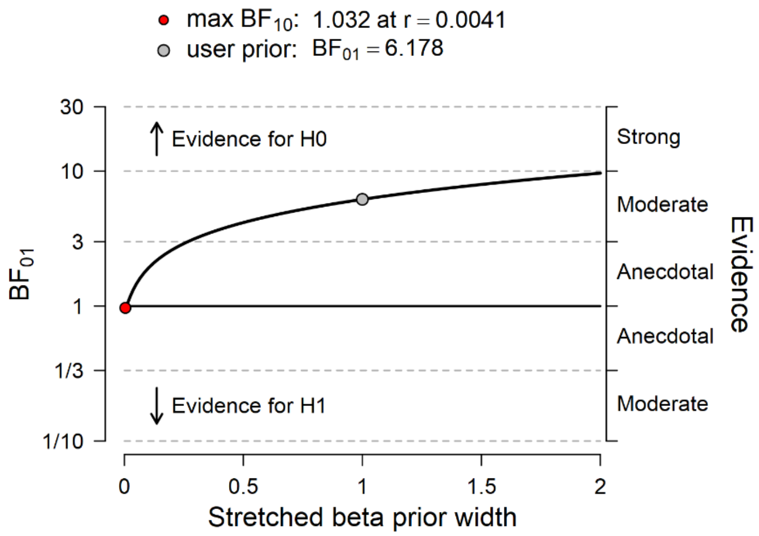

Supplementary Figure 3. Bayes Factor robustness check on the correlation between financial risk-taking and autistic traits.

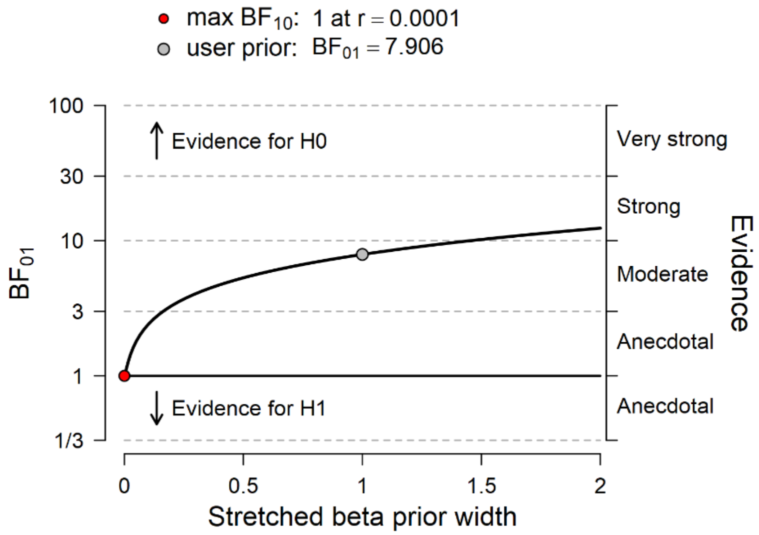

Supplementary Figure 4. Bayes Factor robustness check on the correlation between recreational risk-taking and autistic traits.

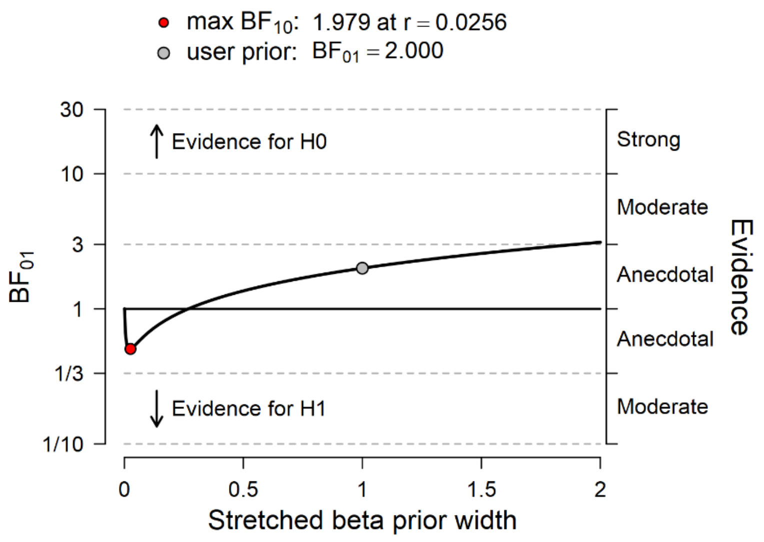

Supplementary Figure 5. Bayes Factor robustness check on the correlation between health/safety risk-taking and autistic traits.

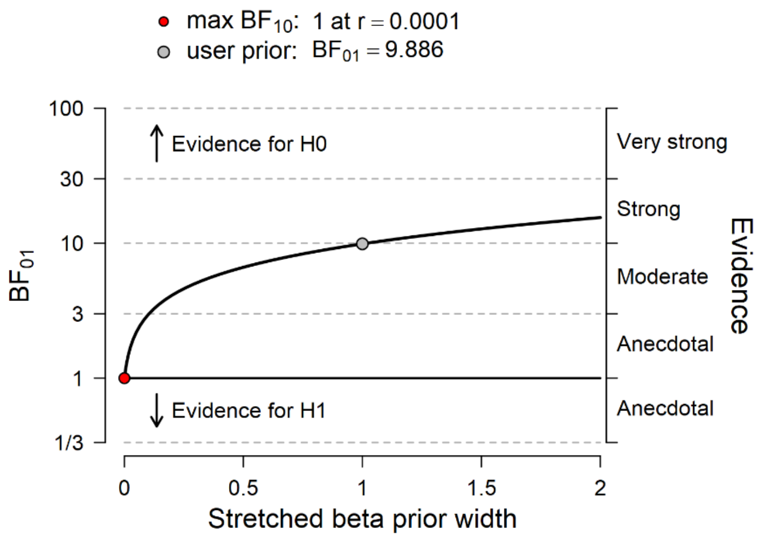

Supplementary Figure 6. Bayes Factor robustness check on the correlation between full scale risk-taking and autistic traits.

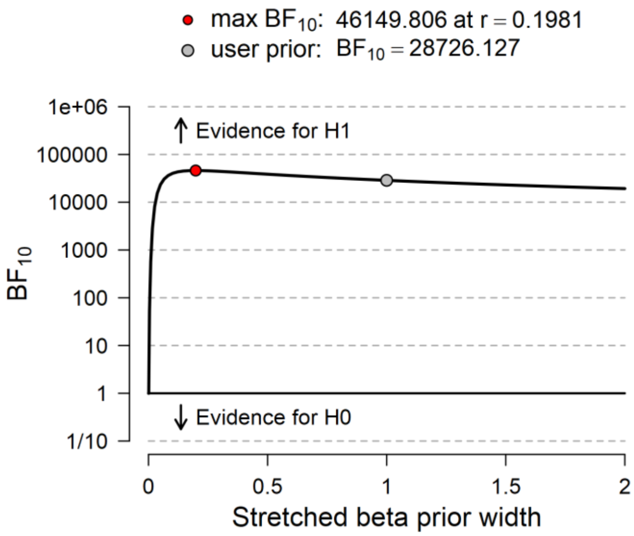

Supplementary Figure 7. Bayes Factor robustness check on the correlation between social risk perception and autistic traits.

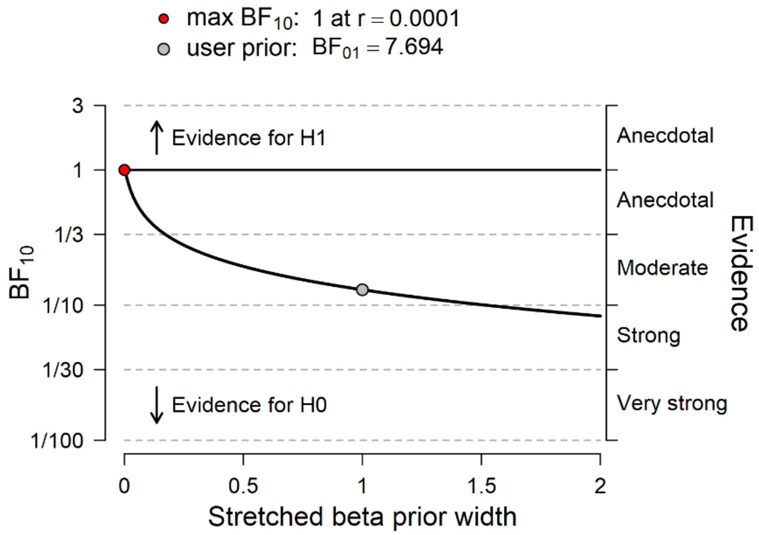

Supplementary Figure 8. Bayes Factor robustness check on the correlation between ethical risk perception and autistic traits.

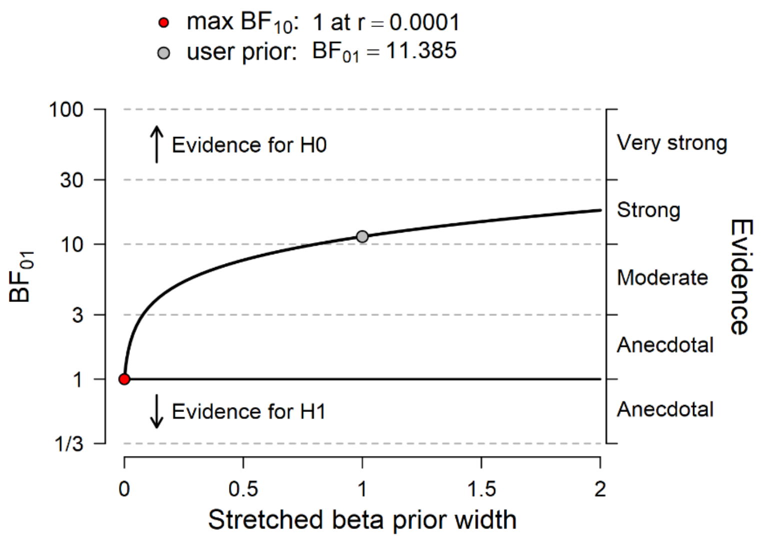

Supplementary Figure 9. Bayes Factor robustness check on the correlation between financial risk perception and autistic traits.

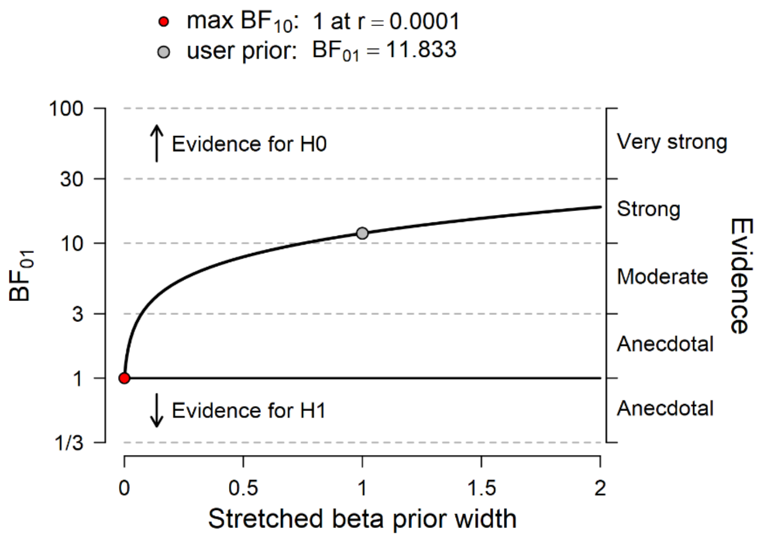

Supplementary Figure 10. Bayes Factor robustness check on the correlation between recreational risk perception and autistic traits.

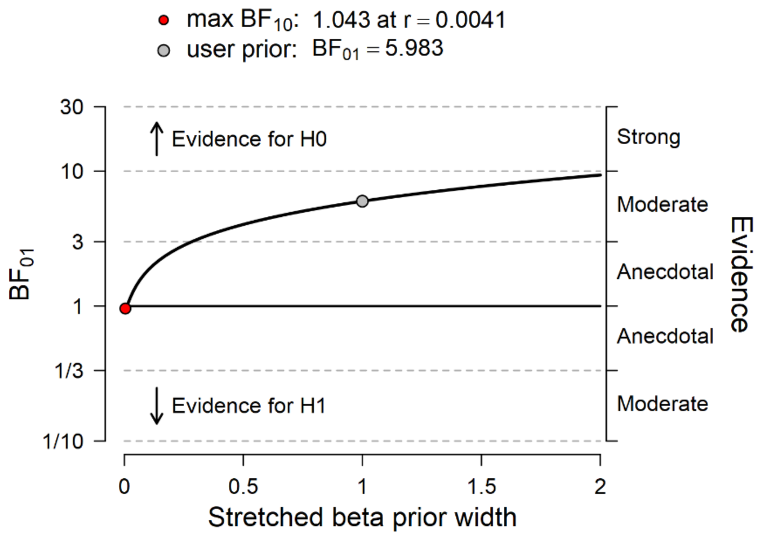

Supplementary Figure 11. Bayes Factor robustness check on the correlation between health/safety risk perception and autistic traits.

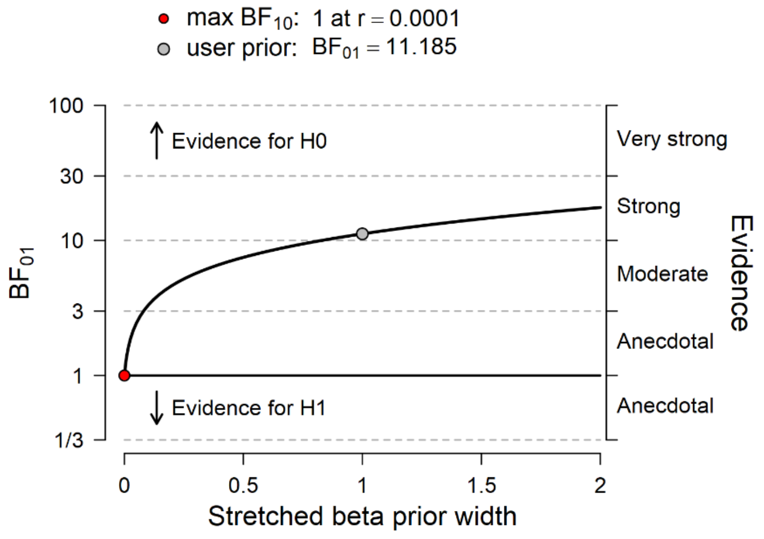

Supplementary Figure 12. Bayes Factor robustness check on the correlation between full scale risk perception and autistic traits.

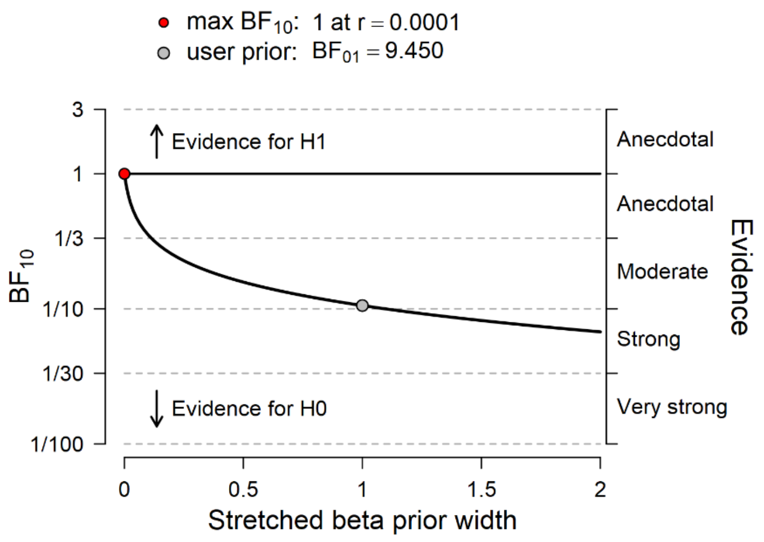

Supplementary Figure 13. Bayes Factor robustness check on the correlation between social expected benefit and autistic traits.

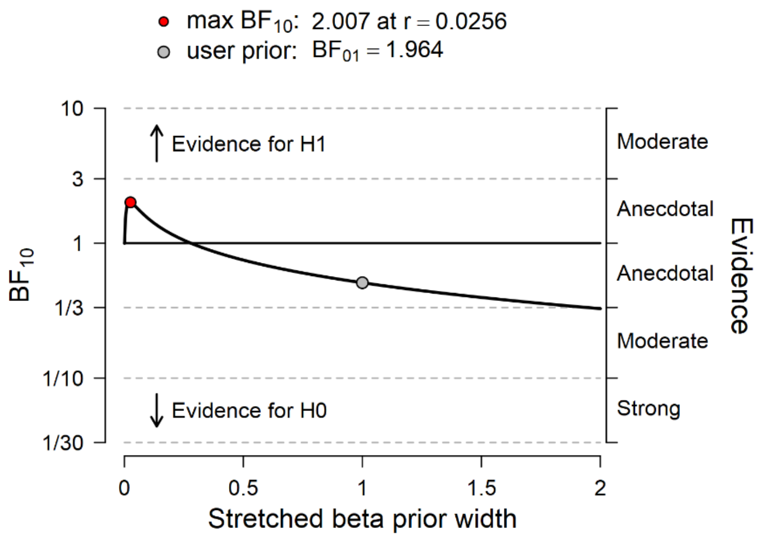

Supplementary Figure 14. Bayes Factor robustness check on the correlation between ethical expected benefit and autistic traits.

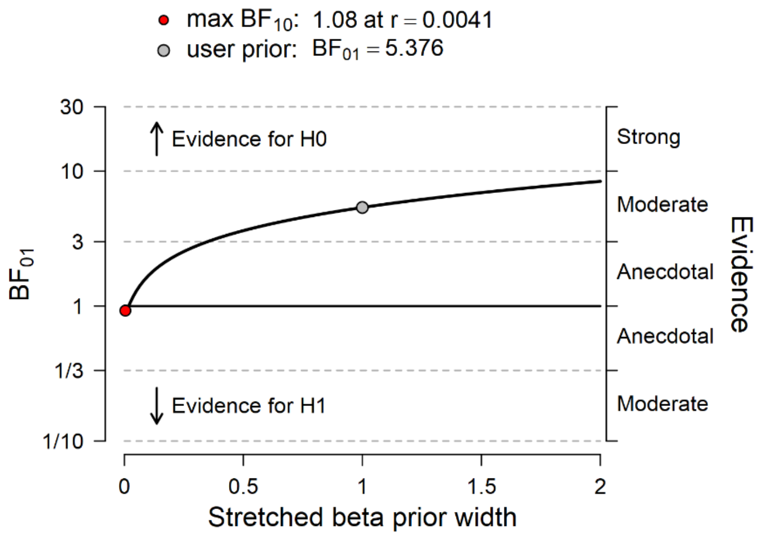

Supplementary Figure 15. Bayes Factor robustness check on the correlation between financial expected benefit and autistic traits.

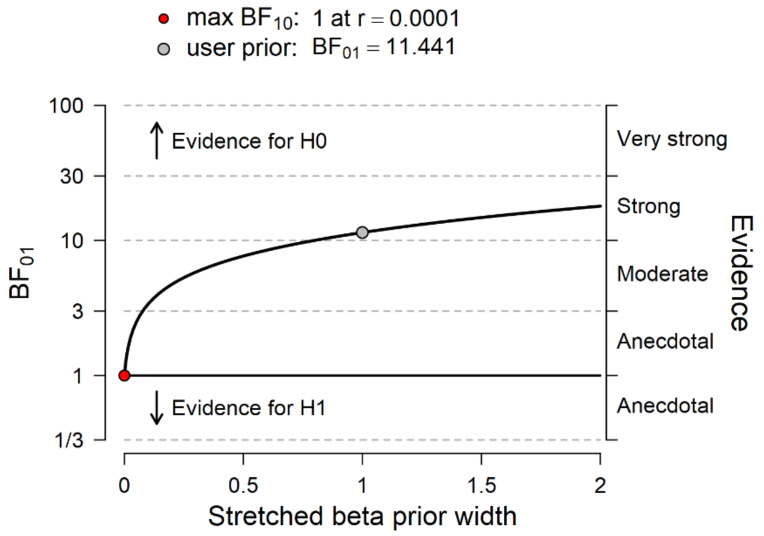

Supplementary Figure 16. Bayes Factor robustness check on the correlation between recreational expected benefit and autistic traits.

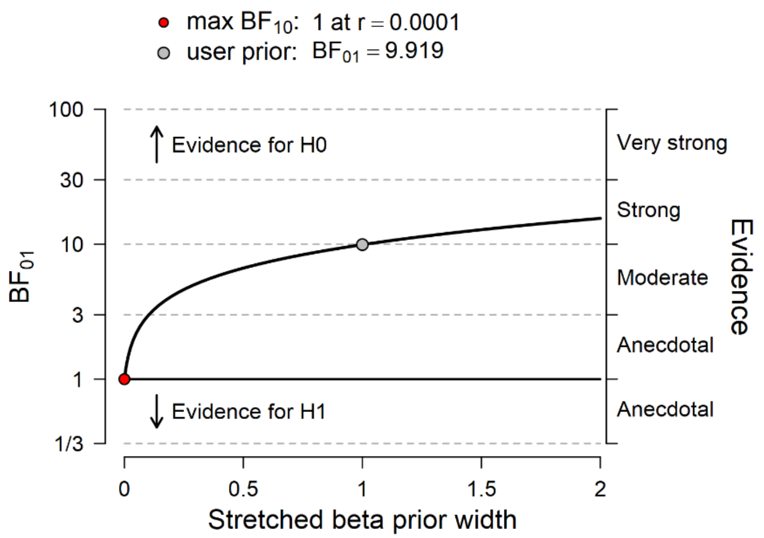

Supplementary Figure 17. Bayes Factor robustness check on the correlation between health/safety expected benefit and autistic traits.

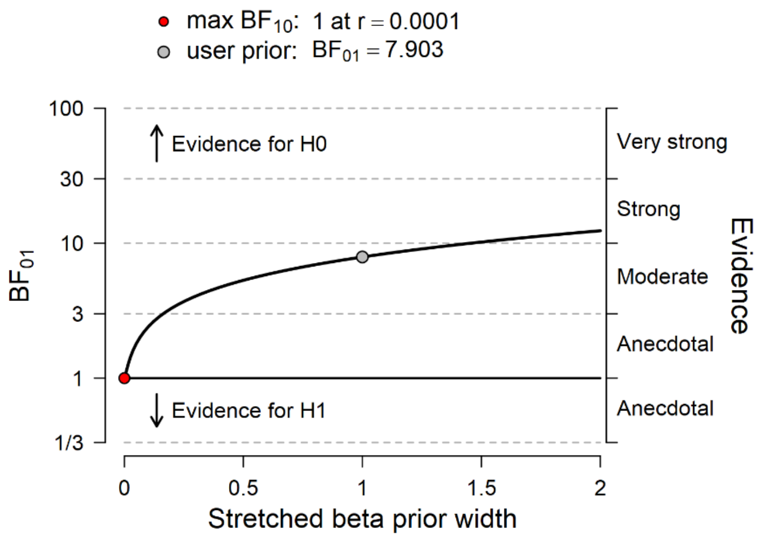

Supplementary Figure 18. Bayes Factor robustness check on the correlation between full scale expected benefit and autistic traits.

2 Robustness check – Impact of Excluding Participants with Current Psychiatric or Neurological Disorders

Supplementary Table 1. Correlations between autistic traits (AQ) and the three DOSPERT scales for each subscale of the DOSPERT separately, after excluding participants with current psychiatric or neurological disorders.

				**Correlation**	
			AQ, DOSPERT Risk-Taking	AQ, DOSPERT Risk Perception	AQ, DOSPERT Expected Benefit
**DOSPERT Subscale**	Social	*r*	-.29	.33	-.01
		BF10	1416.86	34492.82	0.08
	Ethical	*r*	.23	-.06	.15
		BF10	39.25	0.13	0.93
	Financial	*r*	.08	-.03	.08
		BF01	5.99	11.16	5.43
	Recreational	*r*	-.03	-.01	<|.01|
		BF01	10.60	11.78	11.98
	Health/safety	*r*	-.14	-.07	.06
		BF01	1.37	7.24	8.15
	Total	*r*	-.03	.04	.09
		BF01	10.55	10.23	4.85

3 Robustness check – Impact of Conceptual Overlap Between Items from the AQ and DOSPERT

Supplementary Table 2. Correlations between autistic traits (AQ) and the three DOSPERT scales for each subscale of the DOSPERT separately, after correcting for conceptual overlap between items from the AQ and DOSPERT.

			Correlation		
			AQ_{min_social}, DOSPERT Risk-Taking	AQ_{min_social}, DOSPERT Risk Perception	AQ_{min_social}, DOSPERT Expected Benefit
**DOSPERT Subscale**	Social	*r*	-.25	.29	-.03
		BF10	134.72	2647.94	0.09
	Ethical	*r*	.26	-.07	.13
		BF10	280.02	0.15	0.64
	Financial	*r*	.12	-.04	.12
		BF01	2.05	9.81	2.10
	Recreational	*r*	.01	-.05	-.01
		BF01	12.12	9.18	12.31
	Health/safety	*r*	-.07	-.10	.07
		BF01	7.23	3.66	7.40
	Total	*r*	.03	<|.01|	.10
		BF01	10.91	12.35	4.14
